# Supplementary material for: Factors Associated with Depression and Anxiety in Adults ≥60 Years Old during the COVID-19 Pandemic: A Systematic Review
Source: Int J Environ Res Public Health. 2021 Nov 12;18(22):11859. doi: 10.3390/ijerph182211859 (PMC8621514; doi:10.3390/ijerph182211859)
Supplement: Supplementary file 1 [file ijerph-18-11859-s001.zip › ijerph-1445949-supplementary.pdf]

Supplementary Material – Search strategy

Pubmed 23/02/2021

("COVID-19"[Mesh] OR "Coronavirus"[Mesh] OR "SARS-CoV-2"[Mesh] OR "Covid-19" OR "novel coronavirus" OR "sars-cov-2" OR "covid") AND ("Depressive Disorder"[Mesh] OR "Depression"[Mesh] OR "Anxiety"[Mesh] OR "Mental Health"[Mesh] OR "Mental Disorders"[Mesh] OR "anxiety" OR "Anxiety disorder*" OR "depression" OR "depressive symptoms" OR "depressive disorder*") AND ("Aged"[Mesh] OR "Aged population" OR "Aged People" OR "Elderl*" OR "Old people" OR "old population" OR "old adult*" OR "Older people" OR "older population" OR "older adult*")

EMBASE 23/02/2021

('coronavirus disease 2019'/exp OR 'severe acute respiratory syndrome coronavirus 2'/exp OR 'covid-19' OR 'novel coronavirus' OR 'sars-cov-2') AND ('mental health'/exp OR 'mental disease'/exp OR 'depression'/exp OR 'anxiety'/exp OR 'anxiety disorder'/exp OR 'anxiety' OR 'anxiety disorder*' OR 'depression' OR 'depressive symptom*') AND ('aged'/exp OR 'elderl*' OR 'aged people' OR 'aged population' OR 'older adult*' OR 'old adult*' OR 'old people' OR 'older people' OR 'older population' OR 'old population') AND [embase]/lim

Scopus 23/02/2021

TITLE-ABS-KEY (("SARS-CoV-2" OR "Covid-19" OR "novel coronavirus" OR "covid") AND ("Mental Health" OR "Mental Disorders" OR "Anxiety" OR "Anxiety disorder*" OR "Depression" OR "Depressive symptoms" OR "depressive disorder*") AND ("Older adult*" OR "Old adult*" OR "Old people" OR "Older people" OR "Elderl*" OR " Old population" OR " Older population" OR "aged population" OR "aged people"))

ProQuest- Psychology Database 23/02/2021

(MAINSUBJECT.EXACT("COVID-19") OR MAINSUBJECT.EXACT("Severe acute respiratory syndrome coronavirus 2") OR ("coronavirus") OR ("novel coronavirus") OR

(“covid”)) AND (MAINSUBJECT.EXACT("Mental depression") OR MAINSUBJECT.EXACT("Anxieties") OR MAINSUBJECT.EXACT("Mental health") OR MAINSUBJECT.EXACT("Mental disorders") OR ("anxiety") OR ("depression") OR ("depressive disorder") OR ("depressive symptoms") OR ("anxiety disorder")) AND (MAINSUBJECT.EXACT("Older people") OR (("older adult" OR "older adults")) OR (("old adult" OR "old adults")) OR ("elderl*") OR ("old population") OR ("older population") OR ("old people") OR ("older people") OR ("aged people") OR ("aged population"))

Science Direct 23/02/2021

("COVID-19" OR "Coronavirus" OR "SARS-CoV-2") AND ("Mental health" OR “anxiety” OR “depression”) AND ("Aged" OR "old" OR "elderly")

SciELO 23/02/2021

("COVID-19" OR "Coronavirus" OR "SARS-CoV-2") AND ("Mental Disorders" OR "Mental Health" OR “Anxiety” OR “Depression” OR "Depressive symptoms" OR "Depressive disorder*" OR "Anxiety disorder*") AND ("Aged" OR "Elderly" OR "older adult*" OR "old adult*" OR "old people" OR "older people" OR "old population" OR "older population")

COCHRANE LIBRARY 23/02/2021

#1 MeSH descriptor: [COVID-19] explode all trees

#2 MeSH descriptor: [Coronavirus] explode all trees

#3 MeSH descriptor: [SARS-CoV-2] explode all trees

#4 (Sars-Cov-2):ti,ab,kw OR (novel coronavirus):ti,ab,kw OR (Covid):ti,ab,kw OR (Covid-19):ti,ab,kw

#5 MeSH descriptor: [Depression] explode all trees

#6 MeSH descriptor: [Anxiety] explode all trees

#7 MeSH descriptor: [Depressive Disorder] explode all trees

#8 MeSH descriptor: [Anxiety Disorders] explode all trees

#9 MeSH descriptor: [Mental Health] explode all trees

#10 MeSH descriptor: [Mental Disorders] explode all trees

#11 ("anxiety"):ti,ab,kw OR ("anxiety disorder*"):ti,ab,kw OR ("depression"):ti,ab,kw OR ("depressive disorder*"):ti,ab,kw OR (depressive symptoms):ti,ab,kw

#12 MeSH descriptor: [Aged] explode all trees

#13 (elderl*):ti,ab,kw

#14 (aged population):ti,ab,kw OR (aged people):ti,ab,kw

#15 (Old):ti,ab,kw OR (Older):ti,ab,kw

#16 ("adult*"):ti,ab,kw OR ("population"):ti,ab,kw OR ("people"):ti,ab,kw

#17 #15 AND #16

#18 #1 OR #2 OR #3 OR #4

#19 #5 OR #6 OR #7 OR #8 OR #9 OR #10 OR #11

#20 #12 OR #13 OR #14 OR #17

#21 #18 AND #19 AND #20
